# Integrated network pharmacology and experimental validation to explore the potential pharmacological mechanism of Qihuang Granule and its main ingredients in regulating ferroptosis in AMD

**DOI:** 10.1186/s12906-023-04205-3

**Published:** 2023-11-21

**Authors:** Lu Wang, Canyang Zhang, Long Pang, Yan Wang

**Affiliations:** 1 Department of Ophthalmology, The Second Affiliated Hospital of Guangzhou University of Chinese Medicine, 111DaDe Road, Guangzhou, Guangdong 510120 China; 2 Department of Ophthalmology, The Second Clinical College of Guangzhou University of Chinese Medicine, Guangzhou, 510006 China

**Keywords:** Age-related maculopathy (AMD), Ferroptosis, Oxidative stress, Traditional Chinese medicine formula, Network pharmacology

## Abstract

**Background:**

Qihuang Granule (QHG) is a traditional prescription  that has exhibited potential in safeguarding against age-related maculopathy (AMD). Salvia miltiorrhiza (SM) and Fructus lycii (FL) are the main components of QHG. Ferroptosis, a newly discovered, iron-dependent, regulated cell death pathway, have been implicated in the pathogenesis of AMD. This study delves into the intricate mechanism by which SM/FL and QHG confer protection against AMD by modulating the ferroptosis pathway, employing a combination of network pharmacology and experimental validation.

**Methods:**

Bioactive compounds and potential targets of SM and FL were gathered from databases such as TCMSP, GeneCard, OMIM, and FerrDb, along with AMD-related genes and key genes responsible for ferroptosis regulation. Gene ontology (GO), Kyoto Encyclopedia of Genes and Genomes (KEGG) enrichment analysis and protein–protein interaction (PPI) network were performed to discover the potential mechanism. The construction of an interaction network involving AMD, ferroptosis, SM/FL potential target genes was facilitated by the STRING database and realized using Cytoscape software. Subsequent validation was accomplished through molecular docking and in vitro cell experiments.

**Results:**

Noteworthy active compounds including quercetin, tanshinone IIA, luteolin, cryptotanshinone, and hub targets such as HIF-1α, EGFR, IL6, and VEGFA were identified. KEGG enrichment unveiled the HIF-1 signalling pathway as profoundly enriched, and IL6 and VEGF were involved. The molecular docking revealed the significant active compounds with hub genes and quercetin showed good binding to HIF-1α, which is involved in inflammation and angiogenesis. Experimental results verified that both herbs and QHG could regulate key ferroptosis-related targets in the retinal pigment epithelium and inhibit the expression of HIF-1α, VEGFA, and IL-6, subsequently increase cell viability and decrease the ROS content induced by H_2_O_2_.

**Conclusion:**

This study demonstrates the molecular mechanism through which SM/FL and QHG protect against AMD and emerges as a plausible mechanism underlying this protection.

**Supplementary Information:**

The online version contains supplementary material available at 10.1186/s12906-023-04205-3.

## Background

Age-related macular degeneration (AMD) is a prelavent neurodegenerative disease characterized by the progressive deterioration of the macula, which is responsible for sharp and detailed vision. Nowadays, AMD affects more than 50% of people over 80 worldwide. AMD is the primary cause of visual impairment and blindness on a global scale, particularly among the elderly population. Recent statistics reveal a significant prevalence of AMD globally, with an estimated 196 million individuals affected by 2020. The impact of AMD on visual impairment is substantial, accounting for approximately 8.7% of all blindness worldwide. In developed countries, AMD is the leading cause of blindness, particularly among older individuals [1, 2]. AMD not only has a negative impact on individuals' lives but also poses a social and economic burden. Early diagnosis and treatment can effectively reduce vision loss in patients with AMD. Therefore, it is crucial to invest in raising public awareness and strengthening preventive measures as a way to alleviate the economic burden of AMD.

The mechanisms involved in the development of AMD are intricate and multifactorial. These factors include genetic susceptibility, age-related dysfunction of normal retinal homeostasis, impaired lipid metabolism, immune activation leading to chronic inflammation, oxidative stress, and extracellular matrix (ECM) dysfunction [3]. Despite major advances, the exact stochastic relationships among pathogenetic features are largely unknown. Understanding these mechanisms is crucial in preventing the progression of dry AMD to wet AMD and preserving the visual function of patients. Further research is urgently needed to shed light on the underlying mechanisms of AMD and develop effective strategies to halt its progression and minimize visual impairment.

While several tissues are affected in AMD, including photoreceptors, retinal pigment epithelium (RPE), Bruch’s membrane and choriocapillaris, the dysfunction of RPE is an early and crucial event in the molecular pathways that results in clinically relevant changes in AMD patients [4]. In the early stages of AMD, RPE cells susceptible to damage from various factors, leading to impaired function. This impairment reduces their ability to absorb and transport nutrients, resulting in inadequate nutrient supply and subsequent degenerative changes. As damaged RPE cells progressively die, "dry AMD" develops. This cell death triggers an inflammatory response, further harming the surrounding RPE cells and retinal tissue. Additionally, this inflammatory response can stimulate abnormal blood vessel growth, leading to the development of "wet AMD." In summary, the impairment and death of RPE cells play a crucial role in the initiation and progression of AMD [5, 6].

AMD is clinically categorized into early and late stages, with the majority of cases falling into the early stage. This classification includes medium-sized drusen and retinal pigmentary changes. Early-stage AMD is prevalent and imposes a significant economic burden on society [7]. Currently, drug therapies like vitamin C, and vitamin E are commonly employed in the treatment of dry AMD [8]. However, the effectiveness of these therapies for all patients lacks sufficient evidence and outcomes may vary individually. Furthermore, there is currently no specific medication available to cure dry AMD. The primary objective of treatment is to decelerate disease progression and alleviate symptoms.

In recent years, the use of traditional Chinese medicine (TCM) for treating AMD has gained attention as a potential solution. “Qihuang” granule (QHG) is a traditional prescription of TCM and had been studied extensively. It contains compounds such as Salvia miltiorrhiza (SM) and Fructus lycii (FL), which have been found to have protective effects against H_2_O_2_-induced inflammatory injury in human RPE cells and improve visual acuity and fundus conditions in dry AMD patients [9]. In addition, and SM, and FL are commonly used in the treatment of AMD, but the specific mechanisms behind their pharmacological effects are still not fully understood. By delving deeper into the components and mechanisms of action of these Chinese medicines, we can gain a better understanding of their therapeutic effects on age-related macular degeneration. Conducting further studies in this area is crucial as it will provide more scientific evidence for clinical treatment and help patients make informed decisions regarding the use of traditional Chinese medicine for age-related macular degeneration.

Ferroptosis is a recently discovered form of regulated cell death that is dependent on iron and characterized by excessive lipid peroxidation and iron overload. It has been implicated in various conditions such as neurodegeneration, ischaemia–reperfusion injury, and cancer [10–12]. Studies on iron accumulation and elevated lipid peroxidation, and their close association with ferroptosis in the ageing retina have implicated ferroptosis in the pathogenesis of AMD [13]. The activity of the lipid repair enzyme glutathione peroxidase 4 (GPX4) exerts an antioxidative effect on ferroptotic processes, while solute carrier family 7 member 11 (SLC7A11) is part of System Xc-, which regulates ferroptosis together with the glutathione metabolic pathway by exchanging glutamate and cystine [10]. Therefore, the pathways related to GPX4 synthesis and system Xc- function are crucial for the regulation of ferroptosis. Given that ferroptosis is a potential therapeutic target for necroinflammatory diseases, further investigation into its underlying pathophysiological characteristics and molecular mechanisms could provide a basis for developing interventional therapeutic strategies [14].

Network pharmacology is a meaningful approach for drug discovery [15, 16].Our study involves screening effective SM and FL ingredients and analyzing their AMD treatment targets. Additionally, we identified key genes involved in the regulation of ferroptosis. These targets guided us in examining the active ingredient target genes and pathways using network pharmacology, along with gene ontology (GO) and biological pathway (KEGG) functional enrichment analyses. Based on the results, molecular docking technology was used to analyze the optimal effective components from SM, and FL that dock with vital targets to explore the most appropriate compound. By integrating the findings from network pharmacology with in vitro experiments, our study establishes a theoretical foundation for understanding the molecular mechanisms underlying the protective effects of TCMs/FLs and QHG against AMD.

## Methods

### Screening the main active compounds of SM and FL

The ingredients of SM and FL were obtained from the Traditional Chinese Medicine Systems Pharmacology Database and Analysis Platform (TCMSP, https://tcmspw.com/tcmsp.php). TCMSP GeneCards is a comprehensive database integrating information from the Traditional Chinese Medicine Systems Pharmacology (TCMSP) and GeneCards databases. It provides information on the interactions between Chinese herbal compounds and targets, disease-associated genes, and drug targets, helping researchers understand the mechanisms of action and potential pharmacological effects of Chinese herbal compounds. To obtain relevant targets of the main compounds of SM and FL, oral bioavailability (OB) ≥ 30% and drug-likeness (DL) ≥ 0.18 were selected as screening conditions in this study [17]. UniProt database (https://www.uniprot.org/) was chosen to determine the gene name and identifiers of their targets.

### Identification of AMD-, and ferroptosis-related target genes

Potential AMD-related targets were identified through GeneCards [18] (https://www.genecards.org/) and OMIM (Online Mendelian Inheritance in Man, https://www.omim.org/) [19], OMIM (Online Mendelian Inheritance in Man) is a human genetics database that collects information on genetic diseases and related genes. It provides detailed information on disease descriptions, clinical features, inheritance patterns, and the functions of associated genes. The FerrDb database (http://www.zhounan.org/ferrdb/) focuses on genes and proteins related to iron metabolism. It collects information on genes, proteins, and metabolic pathways associated with iron metabolism and related diseases. FerrDb provides detailed data on the functions of iron metabolism genes, expression patterns, and mutations associated with diseases [20]. Overlapping potential target genes of SM/FL between AMD treatment and ferroptosis were acquired through Veeny 2.1 ( https://bioinfogp.cnb.csic.es/tools/venny/) intersection.

### Construction and analysis of a protein–protein interaction (PPI) network

Taking the intersection of all the decoction targets, AMD targets, and ferroptosis targets, we identified the targets that are related to SM/FL-induced ferroptosis in AMD. To further identify the core regulatory targets, PPI analysis was performed by submitting the overlapping targets of active compounds of SM/FL, ferroptosis, and AMD to the STRING biological database (https://string-db.org/) with the species set to *“Homo sapiens”* and the minimum required interaction score was set to 0.40 [21]. Subsequently, the PPI results were exported from STRING and imported into Cytoscape (https://cytoscape.org/, Version 3.7.2), CytoNCA plug-in was used to calculate the parameters, and the hub targets that were related to SM/FL-induced ferroptosis in AMD were identified according to BC, CC, and degree. Finally, the “AMD-Ferroptosis-SM/FL Potential Targets Genes” visual network was constructed with Cytoscape software.

### GO and KEGG enrichment analysis

After transferring the official gene symbols of the identified AMD-Ferroptosis-SM/FL potential target genes to associated Entrez IDs, Gene Ontology (GO) enrichment analysis and Kyoto Encyclopedia of Genes and Genomes (KEGG) pathway enrichment analysis were carried out to further study the functions of the identified potential anti-AMD target genes based on R 4.0.2 and related R packages (colorspace, stringi, DOSE, clusterProfiler, ggplot2, enrichplot, pathview, BiocManager, and org.Hs.eg.db). Only functional terms and pathways with p values < 0.05 were considered statistically significant and retained.

### Analysis of binding capacity between active ingredients and key target genes by molecular docking

Molecular docking is a widely employed method in drug discovery due to its capacity to accurately predict the conformation of small molecule ligands within their appropriate target binding sites and to assess the binding affinity [22–24]. In the present study, the main active compounds and hub genes of SM/FL-induced ferroptosis in AMD were molecularly docked. Candidate target proteins were selected with the following criteria: (1) proteins of human species, (2) with high degree values in the core PPI network, (3) associated with more significant bioactive compounds in the network. The docking process combined AutoDock tools 1.5.6 with Vina (ie, AutoDock Vina), is a novel strategy that has been shown to improve the speed and accuracy of molecular docking with a new scoring function, efficient optimization, and multithreading [25].

### Experimental validation in vitro

#### Cell culture

Human RPE cells (ARPE-19, CL-0026, Procell Life Science & Technology, Wuhan, China) were cultured in DMEM (10566–016, Thermo Fisher Scientific, Waltham, USA), supplemented with 10% foetal bovine serum (1027–106, Thermo Fisher Scientific), 100 U/mL penicillin and 50 U/mL streptomycin (PS2004HY, TBD, Tian Jin, China) at 37 ℃ in 5% CO_2_. The cells showed obvious extension and the density was between 70 and 90%, indicating that the cells were in good condition. This research has been reviewed by the Ethics Committee of Guangdong Provincial Hospital of Chinese Medicine, and it is believed that the project meets the requirements for exemption from ethical review and agrees to exempt the project from review.

To resemble AMD pathophysiology in ARPE-19 cells, we induced oxidative stress with H_2_O_2_ (200 μM, 7722–84-1, Weng Jiang Reagent, Shaoguan, China). To examine the effect of SM/FL or QHG on RPE cells undergoing oxidative stress, cells were plated in 96-well plates at a density of 5 × 10^3^ cells per well and preincubated with 2 g/l SM/FL or 2 g/l QHG for 24 h. Then the cells were further incubated with H_2_O_2_ (200 μm) to induce oxidative stress. The cells were divided into four groups: control group (without drug), H_2_O_2_ group, H_2_O_2_ + SM/FL group and H_2_O_2_ + QHG group.

#### Cell viability assay

RPE cells were preincubated with SM/FL or QHG for 24 h, and then the cells were further incubated with H_2_O_2_ (200 μm) for 12, 24, and 48 h. Cell viability was analyzed with CCK-8 method (C0039, Beyotime Biotechnology, Shanghai, China) in accordance with the manufacturer’s protocol. After treatment, cells were harvested and incubated with 10 μl of CCK-8 solution for 2 h at 37℃. The absorbance was measured at 450 nm with a microplate reader (Multiskoun, Thermo Fisher Scientific), and the optical density represents the proliferation of RPE cells.

#### Western blotting analysis

RPE cells were preincubated with SM/FL or QHG, and then incubated with H_2_O_2_ (200 μm) for 6 h. Protein expression of HIA-1α, VEGFA, IL6, SLC7A11, and GPX4 in each group was measured by Western blotting. Cells were washed with cold PBS, harvested in RIPA buffer (P0013B, Beyotime Biotechnology), incubated on ice for 20 min and centrifuged for 5 min at 500 rpm. Then, protein concentration was quantified using a BCA protein assay kit (P0010, Beyotime Biotechnology). Proteins were separated by SDS-PAGE (S8010, Beijing Solarbio Science & Technology. Beijing, China) and transferred to PVDF membranes by a Trans-Blot Turbo Transfer System. Membranes were blocked with 5% skim milk in for 1 h and incubated with the following primary antibodies overnight at 4 °C: polyclonal rabbit antihuman HIA-1α (1:500, A11945, ABclonal, Wuhan, China), polyclonal rabbit antihuman VEGFA (1:500, ET1604-28, HUABIO, Hangzhou, China), polyclonal rabbit antihuman IL-6(1:500, BS-6309R, Bioss, Beijing, China), polyclonal rabbit antihuman GPX4 (1:500, FNab03622, Wuhan Fine Biotech, Wuhan, China), polyclonal rabbit antihuman SLC7A11(1:500, FNab10533, Wuhan Fine Biotech) and anti-β-actin(1:500; BM0627, BOSTER, Wuhan, China). Protein bands were visualized by incubation with an anti-rabbit secondary antibody (BA1055, 1:3000; BOSTER) or anti-mouse secondary antibody (BA1051, 1:3000; BOSTER) and chemiluminescence substrates (ECL Plus; TIANGEN, Beijing, China). Finally, the bands were quantified by Image Lab software (Media Cybernetics, Silver Spring, Maryland, USA).

#### Measurements of ROS levels

ARPE-19 cells with different treatment were incubated with H_2_O_2_ (200 μm) for 6 h and cellular ROS levels were measured by dihydroethidium (DHE) staining. The cells were washed with PBS twice and diluted, and 10 mM of DHE (S0033S, Beyotime Biotechnology) was incubated with the cells for 30 min at 37 °C, after which cells were washed with PBS again. ROS production was observed with a fluorescence microscope (AE30/31, Nikon, Shanghai city, China). ImageJ software was applied to quantify and calculate the fluorescence intensity.

#### Statistical analysis

All the experiments were performed at least three times. The experimental data were statistically analyzed using GraphPad Prism software and SPSS20.0 Software. Single-factor ANOVA was applied to assess differences in experimental data between groups. *p-value* less than *0.05* were considered as statistically significant.

## Result

### Compound identification and target prediction

**Fig. 1:**
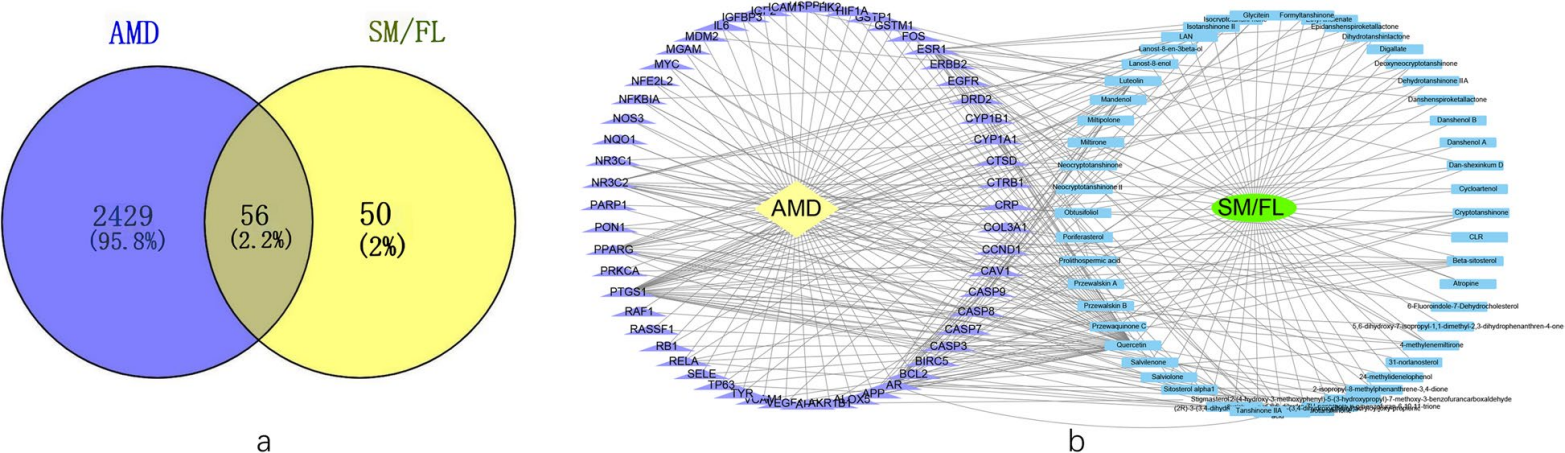
Identification of the drug-disease interaction. **a** The Venn diagram illustrates the targets genes of the two herbs and AMD. **b** The intersection of identified target genes of active compounds in SM/FL and AMD in the Venn diagram. The purple circle represents the targets of disease and the bule circle represents the targets of active compounds of SM/FL

SM and FL are the primary components of QHG, and information about these components is presented in Table 1. According to the active ingredient screening thresholds, 109 active compounds were identified from TCMSP database (Supplementary 1), including 65 compounds from SM and 44 compounds from FL. A total of 106 reviewed or predicted target genes of the bioactive compounds in SM/FL were retrieved from the Uniprot database after eliminating duplicate values and converting protein names to gene symbols. GeneCard and OMIM databases were searched, yielding a total of 2485 AMD target genes. After combining the AMD-related targets and SM/FL-related targets, 56 overlapping targets were recognized as common genes (Fig. 1a). The results of compound-disease target network analysis are showed in Fig. 1b. The link between two circles represents the association of the targets. The top eight active compounds corresponding to the intersection of disease and SM/FL targets are listed in Table 2.

**Table 1:** Information of components in Qihuang Granule (QHG)

Botanical name	Herbal name	Chinese name	Voucher no	Ratio
*Salvia miltiorrhiza Bunge*	Radix salvia miltiorrhiza	Dan Shen	SCM20170631	2
*Lycium barbarum L*	Fruit lyceum barbarum	Gou Qi	SCM20177696	1
*Leonurus cardiaca L*	Fructus leonuri	Chong Wei Zi	SCM20173782	1
*Broussonetia kaempferi Siebold*	Fructus broussonetiae	Chu Shi Zi	SCM20172066	1

**Table 2:** Top eight active compounds corresponding to the intersection of SM/FL and AMD targets

Active ingredient	Count
Quercetin	48
Luteolin	19
Tanshinone VI	9
Tanshinone iia	8
Beta-sitosterol	7
Oxy-propionic acid	6
4-methylenemiltirone	5
Cryptotanshinone	5

### Construction and analysis of the SM/FL-Ferroptosis-AMD target gene network

**Fig. 2:**
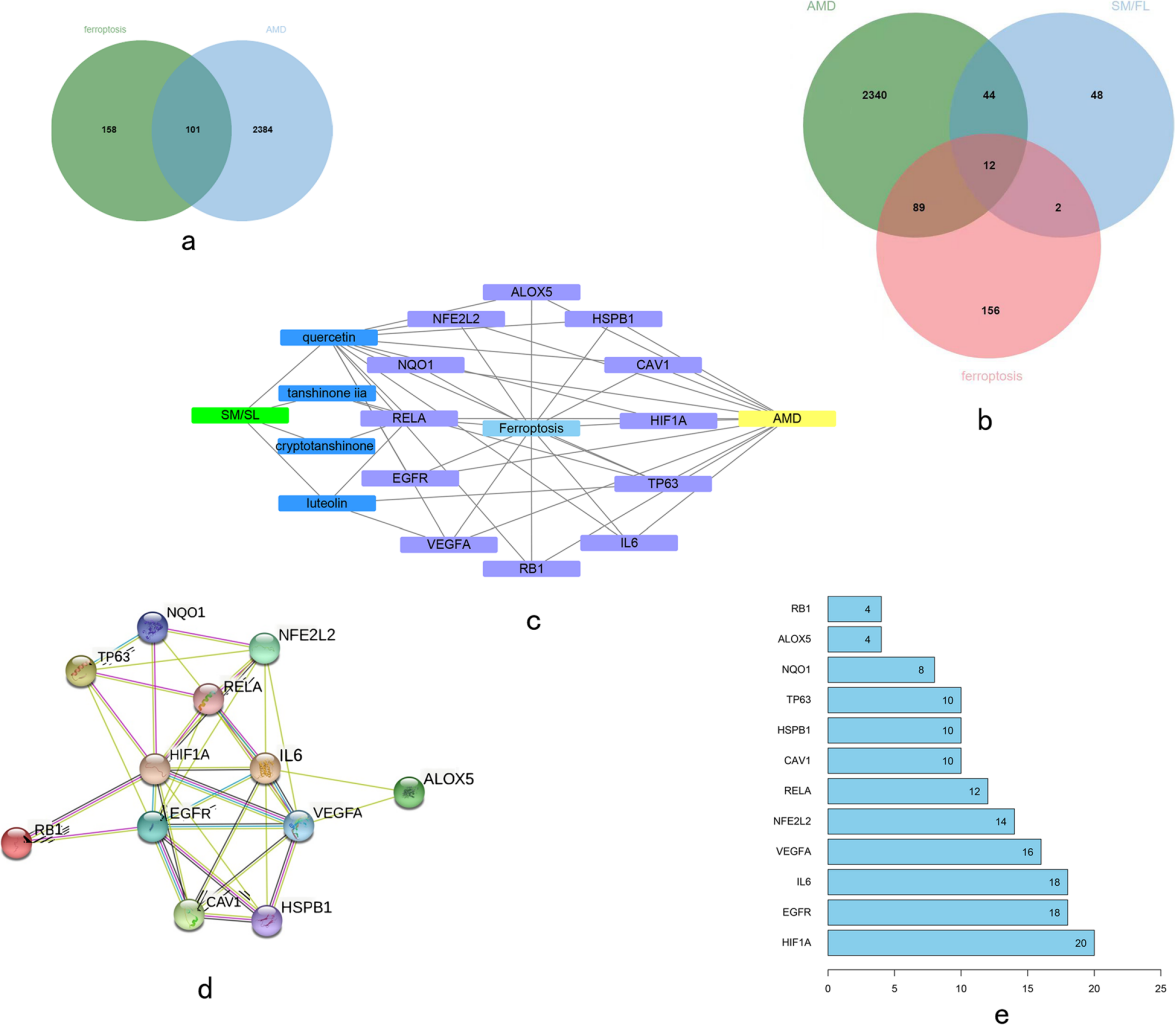
Construction of AMD-Ferroptosis-SM/FL target gene network. **a** The Venn diagram revealed the intersection of target genes of AMD and ferroptosis; (**b**) The Venn diagram shows the AMD-Ferroptosis-SM/FL target gene network; (**c**) Important targets in the protein–protein interaction (PPI) network; (**d**) The PPI network of the AMD-Ferroptosis-SM/FL Target Gene; (**e**) The degree of 12 core targets in the PPI network in **D**

From the FerrDb database, we extracted 259 ferroptosis-associated targets. The overlapping targets of ferroptosis and AMD are shown in a Venn diagram in Fig. 2a. Combining the three target sets, we identified 12 overlapping targets as hub targets for further study (Fig. 2b). Additionally, an AMD-Ferroptosis-SM/FL potential target network was constructed, in which quercetin, tanshinone IIA, luteolin, and cryptotanshinone were considered to be the main active components. The associations among the potential targets of AMD-Ferroptosis-SM/FL are shown in Fig. 2c. According to the degree value, a PPI network was constructed (Fig. 2d). Top five gene nodes were *HIF-1α, EGFR*, *IL6*, *VEGFA*, and *NFE2L2*, with degree values of 20, 18, 18, 16, and 14, respectively (Fig. 2e). These top compounds and genes play important role in the network.

### GO and KEGG pathway enrichment analysis

**Fig. 3:**
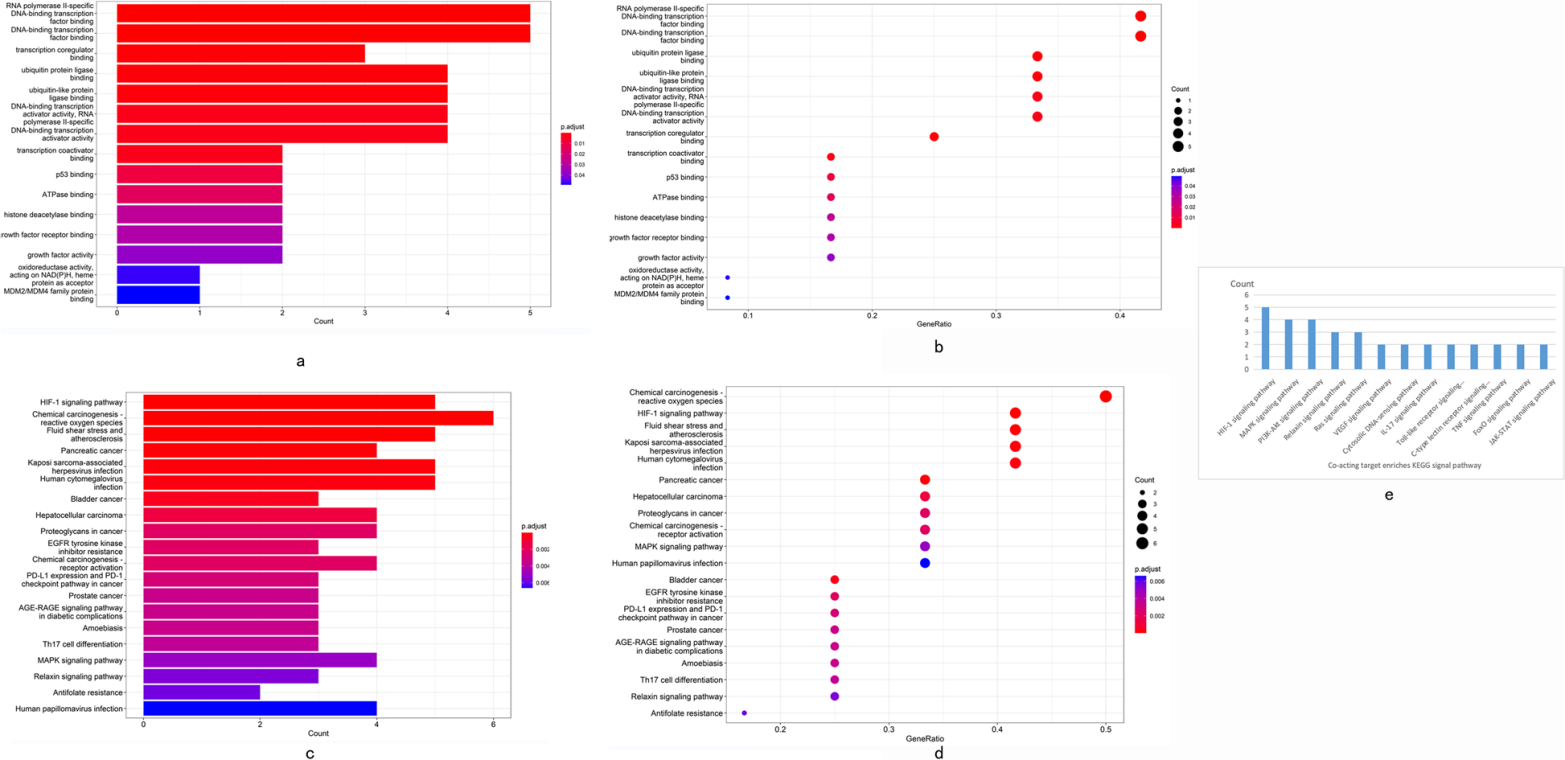
The diagram for GO and KEGG enrichment analysis. **a**, **b** The GO enrichment analysis; (**c**, **d**) The KEGG pathway enrichment analysis. **b**, **d** The abscissa represents the proportion of genes of interest in the entry, and the ordinate represents each entry. The larger size of a dot indicates the larger number of genes annotated in the entry, and the redder color of a dot stands for the lower the p value. **e** The top 13 remarkably enriched KEGG analysis for the signaling pathway of potential target genes

We prepared a file of the overlapping targets of SM/FL-ferroptosis-AMD genes and then used the Bioconductor package to perform a GO enrichment analysis*(p* < *0.05)*. A total of 12 core GO terms were enriched in the biological processes. These included RNA poly II-specific DNA-binding transcription factor binding, DNA-binding transcription factor binding, ubiquitin protein ligase binding, ubiquitin-like protein ligase binding, among others (Fig. 3a, b). Furthermore, enriched KEGG pathways were used to explore the mechanism and signaling pathways related to the SM/FL-ferroptosis pathway in the treatment of AMD. A total of 13 statistically significant signaling pathways were enriched (Fig. 3c, d). Notably, we focused on pathways other than those related to tumors. The top five pathways with the highest gene counts were the HIF-1 signaling pathway, MAPK signaling pathway, P13K-Akt signaling pathway, Relaxin signaling pathway, and Ras signaling pathway. These pathways could potentially mediate protective effects against AMD by reducing ferroptosis (Fig. 3e).

### Molecular docking analysis

**Fig. 4:**
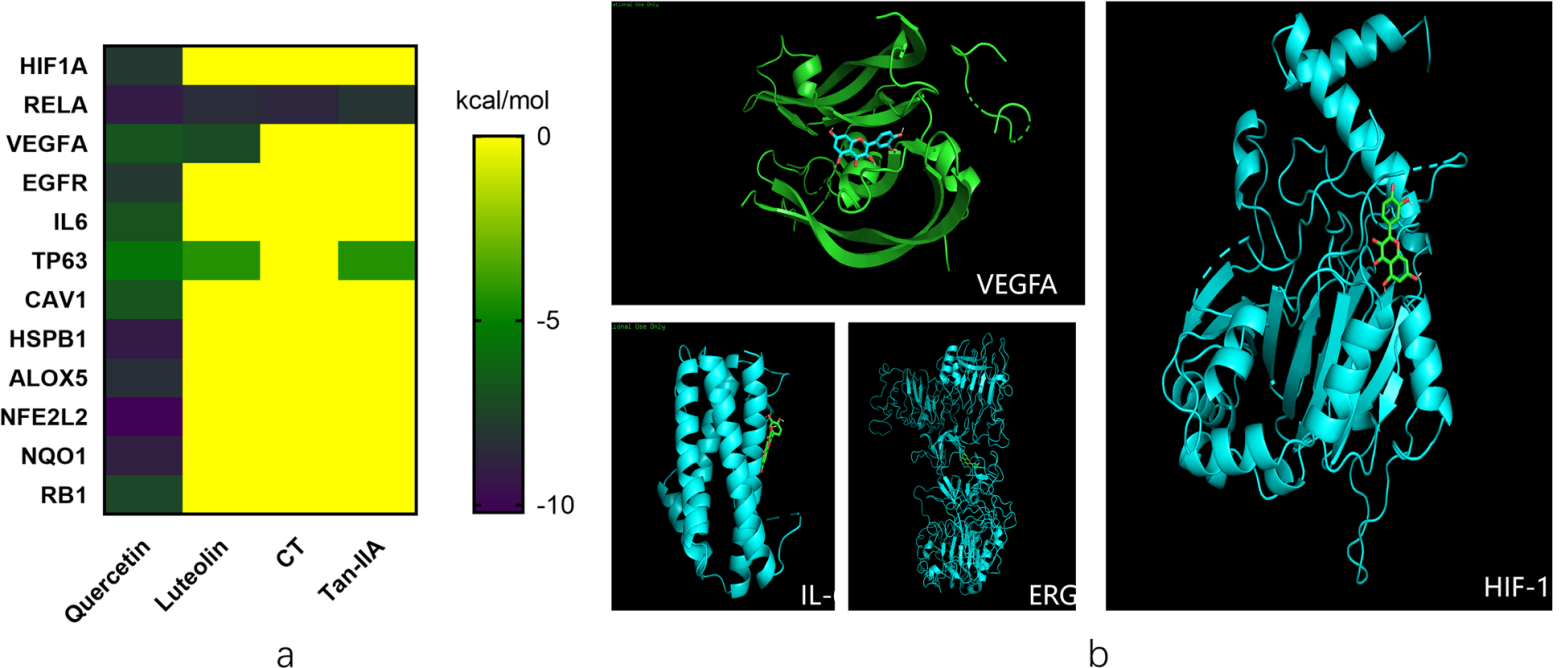
Molecular docking analysis. **a** Heatmap of binding between main active components and hub targets. The darker the color, the better the binding activity. CT: crytotanshinone; tan-IIA: tanshinone IIA; (**b**) Diagram of the docking between key quercetin and core target proteins

Molecular docking analysis was conducted to explore potential direct interactions between SM/FL and target proteins. The main active components, namely, quercetin, tanshinone IIA, luteolin, and crytotanshinone and the main hub genes were studied. A binding energy < -5 kcal mol^−1^ indicates good binding activity (Fig. 4a). Quercetin bound well to all the hub genes, especially HIF-1α, VEGFA, IL6, RELA and ERGF et al. (Fig. 4b). However, tanshinone IIA, luteolin and crytotanshinone bound well only to RELA. Consequently, our focus narrowed to molecular docking analyses of HIF-1α and key targets within the HIF-1 pathway, namely VEGFA and IL6, for further exploration.

### Experimental validation in vitro

#### Pretreatment of RPE Cells with SM/FL or QHG attenuated oxidative stress-induced cytotoxicity

**Fig. 5:**
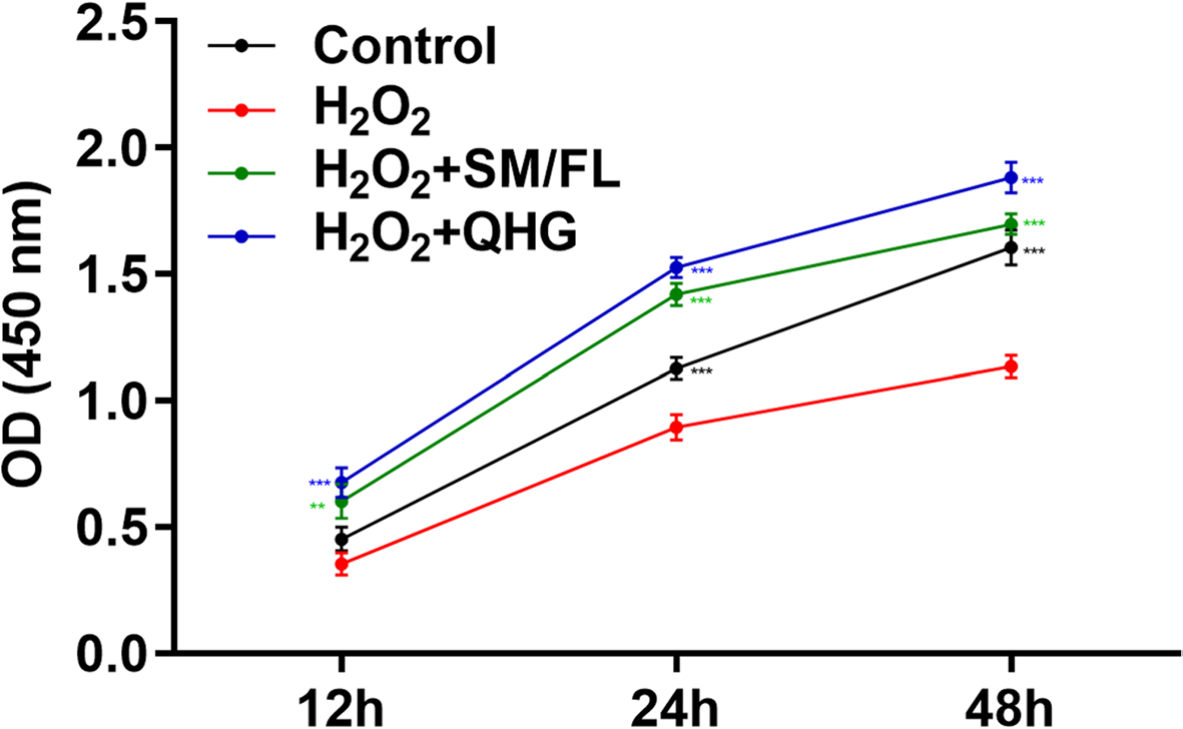
QHG and SM/FL prevented H_2_O_2_ induced loss of cell viability. ARPE-19 cells were pretreated with SM/FL or QHG for 24 h, and then the cells were further incubated with H_2_O_2_ for 12, 24, 48 h and the viability of RPE cells was determined by CCK-8 assay. Data are presented as the means ± SD of 3 independently repeated experiments (* compared H_2_O_2_ group with control, H_2_O_2_ + SM/FL, H_2_O_2_ + QHG group * *p* < 0.05; ** *p* < 0.01 *** *p* < 0.001)

**Fig. 6:**
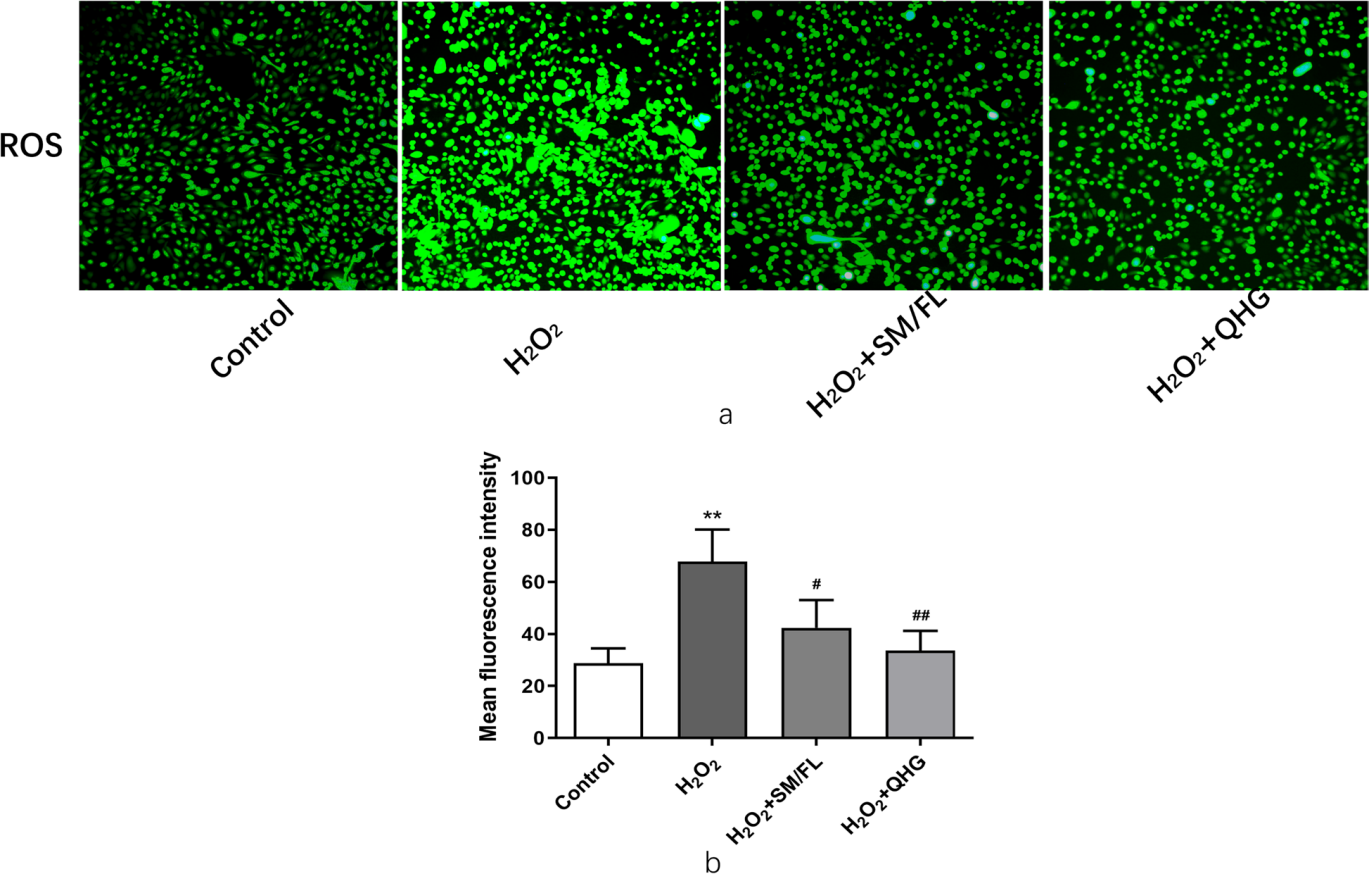
QHG and SM/FL alleviated oxidative stress response induced by H_2_O_2_. ARPE-19 cells were pretreated with SM/FL or QHG for 24 h, and then the cells were further incubated with H_2_O_2_ for 6 h and ROS was detected by Reactive Oxygen Species Assay. **a** Images and (**b**) quantification of dihydroethidium (DHE) staining for intracellular ROS, the original objective magnification is 10 × . Data are presented as the means ± SD of 3 independently repeated experiments (* compared H2O2 group with control, # compared H_2_O_2_ with H_2_O_2_ + SM/FL, H_2_O_2_ + QHG group *, # *p* < 0.05; **, ## *p* < 0.01 ***, ### *p* < 0.001)

To determine the effect of SM/FL or QHG on RPE cells undergoing oxidative stress, cell viability was measured. As shown in Fig. 5, although there were no detectable effects at 12 h, cell viability of RPE cells with treatment of H_2_O_2_ alone resulted in a dramatic decrease at 24, and 48 h. Conversely, pretreatment with SM/FL significantly improved cell viability after treatment with H_2_O_2_ at all time points. The protective effects were more pronounced with QHG. Furthermore, ROS generation was measured in RPE cells that were subjected to different treatments. The results suggested that ROS levels were significantly increased in the H_2_O_2_ group, while this increase was alleviated in cells that were pretreated with SM/FL or QHG under oxidative stress conditions (Fig. 6a, b) *(p* < *0.05)*.

#### Pretreatment with SM/FL or QHG downregulated the expression of HIF-1α and key proteins in related pathways in RPE cells under oxidative stress conditions

**Fig. 7:**
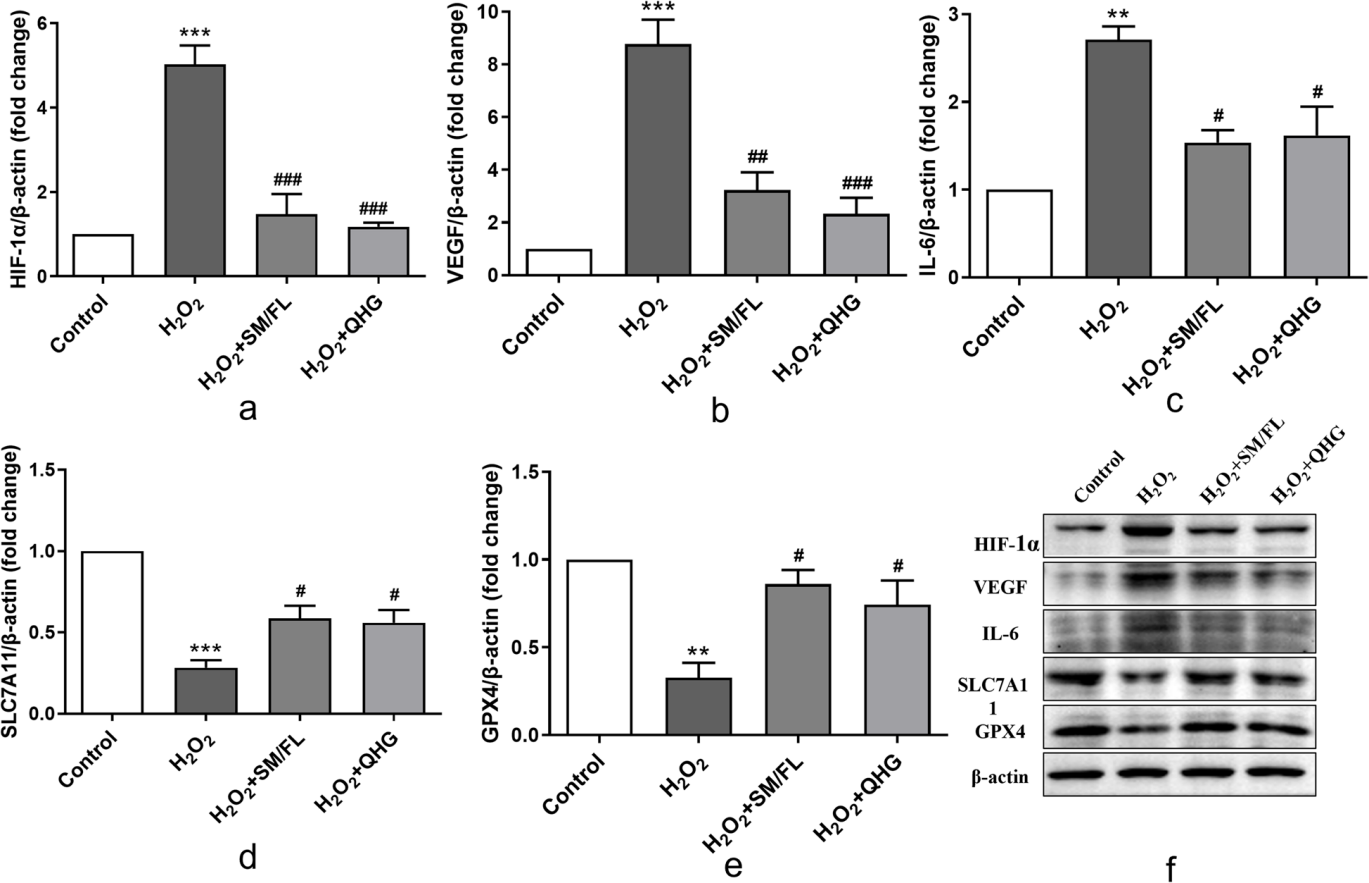
QHG and SM/FL decreased protein levels of HIF-1α/VEGFA/IL6 and increased expression of SLC7A11 and GPX4 proteins. ARPE-19 cells were pretreated with SM/FL or QHG for 24 h, and then the cells were further incubated with H_2_O_2_ for 6 h. Protein levels of HIF-1α (**a**), VEGFA (**b**), IL6 (**c**), SLC7A11 (**d**) and GPX4 (**e**) protein were detected by western blotting (f) and normalized to that of β-actin. Data are presented as the means ± SD of 3 independently repeated experiments. (* compared H_2_O_2_ group with control, # compared H_2_O_2_ with H_2_O_2_ + SM/FL, H_2_O_2_ + QHG group *, # *p* < 0.05; **, ## *p* < 0.01 ***, ### *p* < 0.001)

Increased expression of HIF-1α, VEGFA and IL6 was observed by Western blotting after treatment with H_2_O_2_ alone. In contrast, preincubation with SM/FL or QHG markedly decreased the expression of HIF-1α, VEGFA and IL6 in cells after treatment with H_2_O_2_ (Fig. 7f). Quantification of these results with ImageJ software revealed that the differences were statistically different, and the changes in the protein expression of *HIF-1α* were more pronounced (*p* < *0.05*) (Fig. 7a, b, c).

#### Increased expression of ferroptosis-related key targets in RPE cells that were pretreated with SM/FL or QHG under oxidative stress conditions

We also performed Western blotting analysis to examine the effects of H_2_O_2_, SM/FL and QHG on the expression of the intracellular ferroptosis markers GPX4 and SLC7A11. Compared with RPE cells cultured in DMEM, cells exposed to H_2_O_2._ showed significant downregulation of GPX4 and SLC7A11. Notably, the effect of H_2_O_2_ was almost reversed in RPE cells that were pretreated with SM/FL or QHG (Fig. 7f). The results of Western blotting analysis were also quantified, and SM/FL or QHG significantly increased the expression of GPX4 and SLC7A11 in RPE cells that were preincubated with these TCM agents under oxidative stress conditions. (*p* < *0.05*) (Fig. 7d, e).

## Discussion

Traditional Chinese medicine (TCM) has established its significance in disease prevention and treatment, offering a holistic approach through multiple ingredients that often influence diverse targets and pathways. In our investigation, we harnessed the power of network pharmacology to unravel the potential mechanisms by which Salvia miltiorrhiza (SM) and Fructus lycii (FL), the primary compounds of Qihuang Granule (QHG), exert their protective effects against age-related macular degeneration (AMD). Specifically, we delved into the ferroptosis-related pathway, a pathway that mounting evidence has linked to AMD pathogenesis through its role in inducing oxidative stress-mediated damage and inflammation [13, 26, 27]. Our approach was complemented by in vitro validation experiments, bolstering the robustness of our findings.

Through network pharmacology, we successfully identified 56 shared target genes among bioactive compounds in SM/FL and target genes implicated in AMD. Notably, quercetin, a key component of SM/FL, stood out as the compound with the highest number of associations with AMD-related genes and ferroptosis. This suggests quercetin's paramount role in treating AMD by targeting ferroptosis. Subsequently, we pinpointed 12 core targets through which SM/FL may protect against AMD via ferroptosis regulation. Among these, *HIF-1α, EGFR, IL6, VEGFA, and NFE2L2* emerged as the top five hub targets based on degree values within the protein–protein interaction (PPI) network. Gene ontology (GO) and Kyoto Encyclopedia of Genes and Genomes (KEGG) analyses illuminated these core targets' involvement in biological processes, including RNA poly II-specific DNA-binding transcription factor binding, DNA-binding transcription factor binding, ubiquitin protein ligase binding, and ubiquitin-like protein ligase binding, mediated by the HIF-1, MAPK, P13K-Akt, Relaxin, and Ras signaling pathways.

Molecular docking analysis provided further substantiation by confirming the interaction between active compounds in SM/FL and hub genes. These findings underscore the effectiveness of SM/FL in treating AMD from a bioinformatics perspective, elucidating the underlying mechanisms of QHG's actions. In line with the predictions from network pharmacology, our in vitro experiments revealed that pre-treatment with SM/FL or QHG effectively countered H_2_O_2_-induced declines in cell viability and the increase of HIF-1α, VEGFA, and IL6. Additionally, both SM/FL and QHG elevated the expression of key ferroptosis-related proteins SLC7A11 and GPX4 while mitigating ferroptosis reactions and oxidative stress responses in RPE cells. These outcomes serve as a valuable corroboration of our network pharmacology analyses.

Qi Huang Granule (QHG), a traditional prescription comprising SM and FL as its primary components, has previously demonstrated its efficacy in improving visual acuity and fundus conditions in dry AMD patients. SM, renowned for its antioxidative, neuroprotective, anti-inflammatory, and antineoplastic properties, has been shown to mitigate RPE damage and hypoxia by suppressing inflammation in the retina [28–32]. FL, a fixture in traditional Chinese pharmacopeia for centuries, boasts potent antioxidant effects and the ability to scavenge superoxide anions and hydroxyl radicals, pivotal in reducing oxidative stress [33, 34]. FL extract pre-treatment has exhibited protective effects against acute oxidative stress injuries in human RPE cells, fostering viable cell proliferation, reducing apoptosis, enhancing phagocytic capabilities, and curbing lipofuscin accumulation [33, 35].

Our study pinpointed four active ingredients within SM/FL that play pivotal roles in AMD treatment by regulating ferroptosis, underscoring their potential for further research. Quercetin, the most abundant compound in SM/FL, boasts diverse biological functions such as antioxidant, anti-inflammatory, and anti-viral activities [36–38]. Our PPI research has identified quercetin as a potential mediator of ferroptosis through its ability to interact with multiple hub targets. Studies have shown that quercetin can inhibit ferroptosis in several conditions, including inflammatory, neurodegenerative, and age-related diseases via several pathways [39, 40]. In age-related disease, quercetin can inhibit ferroptosis and inhibit oxidative stress, reduce inflammatory response, and restore mitochondrial function [41]. In AMD, quercetin could protect RPE cells from oxidative damage and cellular senescence in vitro in a dose-dependent manner via the inhibition of proinflammatory molecules, or it could directly inhibit the intrinsic apoptosis pathway [42]. In a mouse model of dry AMD, quercetin also reduced RPE sediments and Bruch’s membrane thickness [43]. Tanshinone IIA (tan-IIA), a major fat-soluble component of Salvia miltiorrhiza, has demonstrated angiogenesis, anti-inflammatory, and antioxidant activities. Tan-IIA can regulate ferroptosis through various pathways and our PPI network revealed that Tan-IIA may impact AMD by targeting *RELA* and *TP63* [44, 45] In RPE cells, Tan-IIA exerts a protective effect against oxidative stress by inhibiting HIF-1α secretion and apoptosis [46, 47].

Luteolin, known primarily for its anti-inflammatory activity, protects RPE cells from oxidative stress-induced cell death, mitigates epithelial-mesenchymal transformation, and suppresses IL-1β-induced adhesion in AMD [48–50]. Cryptotanshinone (CTS), a significant component of SM, has garnered attention primarily in cancer and cardiovascular disease studies [51]. Our pharmacological network analysis unearthed CTS as a potential regulator of RELA, suggesting a novel avenue for CTS research in AMD.

As seen in the AMD-Ferroptosis-SM/FL Potential Targets Genes network, many target genes can be regulated by different active compounds and one active compound can target several genes. These results suggest that this therapy has multicomponent, multitarget biological attributes. Additionally, the PPI results suggest that the 12 target proteins are not independent of each other but are linked and interact. These results also indicate that SM/FL can be involved in the alleviation and treatment of AMD through the regulation of multiple proteins.

HIF-1α emerged as a pivotal hub target, with the HIF-1α pathway ranking first in PPI and KEGG analyses. HIF-1α, a subunit of HIF-1, acts as a master regulator of hypoxia-inducible genes associated with inflammation, angiogenesis, cell proliferation/survival, and glucose/iron metabolism [52]. Notably, HIF-1α's role in ferroptosis regulation has been documented. In diabetic nephropathy, ferroptosis can trigger renal tubule damage through the HIF-1α pathway by upregulating HO-1 expression, leading to iron overload, excessive ROS production, and lipid peroxidation [53]. HIF-1α can also influence other ferroptotic genes such as Tf and Tfrc, impacting SLC7A11 expression [54]. Moreover, HIF-1α's involvement in AMD pathogenesis is well-established, with higher expression levels found in drusen tissue samples from elderly patients [55–58]. Our findings indicated that VEGFA and IL6 are part of the HIF-1α pathway. BIKUL DAS et al. found that, after applying siRNA to block HIF-1A or VEGF in human RPE-19 cell lines, the expression of IL6, IL8, and MCP changed, indicating a relationship between HIF-1A, VEGF and IL6 [59]. Transcriptional activation of *HIF-1α* can upregulate the expression of VEGF in general and in RPE cells. It is thus a central molecule for triggering CNV formation [60] These results are congruent with our results that HIF-1a, VEGF, and IL6 is extremely important and may participate in the process of SM/FL or QHG against AMD by regulating ferroptosis. Our study identified the MAPK pathway's involvement in ferroptosis, with activation of MAPK signaling contributing to ferroptosis in various contexts, including oxidative stress-induced RPE degeneration. These findings open avenues for investigating pharmacological inhibitors targeting the MAPK pathway as a potential combination therapy for AMD [61, 62].

Our in vitro experiments further solidified the connection between SM/FL, QHG, ferroptosis, and AMD. Western blotting demonstrated decreased expression of HIF-1α, VEGFA, and IL6, alongside increased SLC7A11 and GPX4 expression in the SM/FL and QHG groups compared to the H2O2 group. This translates to not only potential therapeutic benefits but also a reduction in proinflammatory factors that exacerbate AMD. The elevated cell viability and stability observed in SM/FL and QHG-treated cells corroborate the network pharmacology findings.

One limitation of this study pertains to the absence of consideration for interactions between active ingredients, as well as the caveat that compound absorption in humans is not solely determined by the oral bioavailability (OB). Further exploration is warranted to elucidate the intricate relationship between HIF-1α, VEGFA, and IL6 in the context of AMD.

## Conclusion

In conclusion, our comprehensive approach, integrating network pharmacological analysis and in vitro experiments, sheds light on the mechanisms that underlie the protective effects of Salvia miltiorrhiza (SM) and Fructus lycii (FL), as well as their composite, Qihuang Granule (QHG), against age-related macular degeneration (AMD). These findings not only advance our understanding of the therapeutic mechanisms of traditional Chinese medicine but also present a promising strategy for deciphering the scientific rationale and therapeutic modalities of traditional Chinese medicine formulas in addressing complex diseases.

### Supplementary Information

**Additional file 1.**

**Additional file 2.**

## Data Availability

All data generated or analysed during this study are included in this published article.

## Abbreviations

QHG: Qihuang Granule
AMD: Age-related macular degeneration
SM: Salvia miltiorrhiza
FL: Fructus lycii
GO: Gene ontology
KEGG: Kyoto Encyclopedia of Genes and Genomes
PPI: Protein-protein interaction
RPE: Retinal pigment epithelium
GPX4: Glutathione peroxidase 4
SLC7A11: Solute carrier family 7 member 11
TCM: Traditional Chinese medicine
