# Supplementary material for: Integrated network pharmacology and experimental validation to explore the potential pharmacological mechanism of Qihuang Granule and its main ingredients in regulating ferroptosis in AMD
Source: BMC Complement Med Ther. 2023 Nov 21;23:420. doi: 10.1186/s12906-023-04205-3 (PMC10664676; doi:10.1186/s12906-023-04205-3)
Supplement: Supplementary file 1 — Additional file 1. [file 12906_2023_4205_MOESM1_ESM.docx]

| **Mol ID** | **Effective component** | **OB(%)** | **DL** |
| --- | --- | --- | --- |
| MOL007064 | przewalskin b | 110.32 | 0.44 |
| MOL007132 | (2R)-3-(3,4-dihydroxyphenyl)-2-[(Z)-3-(3,4-dihydroxyphenyl)acryloyl]oxy-propionic acid | 109.38 | 0.35 |
| MOL009664 | Physalin A | 91.71 | 0.27 |
| MOL007140 | (Z)-3-[2-[(E)-2-(3,4-dihydroxyphenyl)vinyl]-3,4-dihydroxy-phenyl]acrylic acid | 88.54 | 0.26 |
| MOL007150 | (6S)-6-hydroxy-1-methyl-6-methylol-8,9-dihydro-7H-naphtho[8,7-g]benzofuran-10,11-quinone | 75.39 | 0.46 |
| MOL007058 | formyltanshinone | 73.44 | 0.42 |
| MOL007120 | miltionone Ⅱ | 71.03 | 0.44 |
| MOL007105 | epidanshenspiroketallactone | 68.27 | 0.31 |
| MOL007155 | (6S)-6-(hydroxymethyl)-1,6-dimethyl-8,9-dihydro-7H-naphtho[8,7-g]benzofuran-10,11-dione | 65.26 | 0.45 |
| MOL007130 | prolithospermic acid | 64.37 | 0.31 |
| MOL007050 | 2-(4-hydroxy-3-methoxyphenyl)-5-(3-hydroxypropyl)-7-methoxy-3-benzofurancarboxaldehyde | 62.78 | 0.4 |
| MOL007068 | Przewaquinone B | 62.24 | 0.41 |
| MOL000569 | digallate | 61.85 | 0.26 |
| MOL007081 | Danshenol B | 57.95 | 0.56 |
| MOL007082 | Danshenol A | 56.97 | 0.52 |
| MOL007069 | przewaquinone c | 55.74 | 0.4 |
| MOL007108 | isocryptotanshi-none | 54.98 | 0.39 |
| MOL007125 | neocryptotanshinone | 52.49 | 0.32 |
| MOL007079 | tanshinaldehyde | 52.47 | 0.45 |
| MOL007088 | cryptotanshinone | 52.34 | 0.4 |
| MOL008400 | glycitein | 50.48 | 0.24 |
| MOL007094 | danshenspiroketallactone | 50.43 | 0.31 |
| MOL007111 | Isotanshinone II | 49.92 | 0.4 |
| MOL007154 | tanshinone iia | 49.89 | 0.4 |
| MOL007119 | miltionone Ⅰ | 49.68 | 0.32 |
| MOL007098 | deoxyneocryptotanshinone | 49.4 | 0.29 |
| MOL007048 | (E)-3-[2-(3,4-dihydroxyphenyl)-7-hydroxy-benzofuran-4-yl]acrylic acid | 48.24 | 0.31 |
| MOL006209 | cyanin | 47.42 | 0.76 |
| MOL009651 | Cryptoxanthin monoepoxide | 46.95 | 0.56 |
| MOL007051 | 6-o-syringyl-8-o-acetyl shanzhiside methyl ester | 46.69 | 0.71 |
| MOL000098 | quercetin | 46.43 | 0.28 |
| MOL009612 | (24R)-4alpha-Methyl-24-ethylcholesta-7,25-dien-3beta-ylacetate | 46.36 | 0.84 |
| MOL001495 | Ethyl linolenate | 46.1 | 0.2 |
| MOL005406 | atropine | 45.97 | 0.19 |
| MOL007156 | tanshinone Ⅵ | 45.64 | 0.3 |
| MOL007141 | salvianolic acid g | 45.56 | 0.61 |
| MOL001942 | isoimperatorin | 45.46 | 0.23 |
| MOL007101 | dihydrotanshinoneⅠ | 45.04 | 0.36 |
| MOL007115 | manool | 45.04 | 0.2 |
| MOL007123 | miltirone Ⅱ | 44.95 | 0.24 |
| MOL007045 | 3α-hydroxytanshinoneⅡa | 44.93 | 0.44 |
| MOL007449 | 24-methylidenelophenol | 44.19 | 0.75 |
| MOL009665 | Physcion-8-O-beta-D-gentiobioside | 43.9 | 0.62 |
| MOL001659 | Poriferasterol | 43.83 | 0.76 |
| MOL009618 | 24-ethylcholesta-5,22-dienol | 43.83 | 0.76 |
| MOL000449 | Stigmasterol | 43.83 | 0.76 |
| MOL009622 | Fucosterol | 43.78 | 0.76 |
| MOL002651 | Dehydrotanshinone II A | 43.76 | 0.4 |
| MOL009644 | 6-Fluoroindole-7-Dehydrocholesterol | 43.73 | 0.72 |
| MOL007077 | sclareol | 43.67 | 0.21 |
| MOL007142 | salvianolic acid j | 43.38 | 0.72 |
| MOL001323 | Sitosterol alpha1 | 43.28 | 0.78 |
| MOL007152 | Przewaquinone E | 42.85 | 0.45 |
| MOL007151 | Tanshindiol B | 42.67 | 0.45 |
| MOL009641 | 4alpha,24-dimethylcholesta-7,24-dienol | 42.65 | 0.75 |
| MOL009681 | Obtusifoliol | 42.55 | 0.76 |
| MOL009621 | 24-methylenelanost-8-enol | 42.37 | 0.77 |
| MOL009642 | 4alpha-methyl-24-ethylcholesta-7,24-dienol | 42.3 | 0.78 |
| MOL009634 | 31-norlanosterol | 42.2 | 0.73 |
| MOL001979 | LAN | 42.12 | 0.75 |
| MOL001494 | Mandenol | 42 | 0.19 |
| MOL009656 | (E,E)-1-ethyl octadeca-3,13-dienoate | 42 | 0.19 |
| MOL007070 | (6S,7R)-6,7-dihydroxy-1,6-dimethyl-8,9-dihydro-7H-naphtho[8,7-g]benzofuran-10,11-dione | 41.31 | 0.45 |
| MOL007041 | 2-isopropyl-8-methylphenanthrene-3,4-dione | 40.86 | 0.23 |
| MOL009646 | 7-O-Methylluteolin-6-C-beta-glucoside_qt | 40.77 | 0.3 |
| MOL007071 | przewaquinone f | 40.31 | 0.46 |
| MOL002776 | Baicalin | 40.12 | 0.75 |
| MOL009653 | Cycloeucalenol | 39.73 | 0.79 |
| MOL007118 | microstegiol | 39.61 | 0.28 |
| MOL006824 | α-amyrin | 39.51 | 0.76 |
| MOL007124 | neocryptotanshinone ii | 39.46 | 0.23 |
| MOL009660 | methyl (1R,4aS,7R,7aS)-4a,7-dihydroxy-7-methyl-1-[(2S,3R,4S,5S,6R)-3,4,5-trihydroxy-6-(hydroxymethyl)oxan-2-yl]oxy-1,5,6,7a-tetrahydrocyclopenta[d]pyran-4-carboxylate | 39.43 | 0.47 |
| MOL009640 | 4alpha,14alpha,24-trimethylcholesta-8,24-dienol | 38.91 | 0.76 |
| MOL007093 | dan-shexinkum d | 38.88 | 0.55 |
| MOL007122 | Miltirone | 38.76 | 0.25 |
| MOL001601 | 1,2,5,6-tetrahydrotanshinone | 38.75 | 0.36 |
| MOL003578 | Cycloartenol | 38.69 | 0.78 |
| MOL007100 | dihydrotanshinlactone | 38.68 | 0.32 |
| MOL009662 | Lantadene A | 38.68 | 0.57 |
| MOL009631 | 31-Norcyclolaudenol | 38.68 | 0.81 |
| MOL009633 | 31-norlanost-9(11)-enol | 38.35 | 0.72 |
| MOL009639 | Lophenol | 38.13 | 0.71 |
| MOL009620 | 24-methyl-31-norlanost-9(11)-enol | 38 | 0.75 |
| MOL000953 | CLR | 37.87 | 0.68 |
| MOL009635 | 4,24-methyllophenol | 37.83 | 0.75 |
| MOL005438 | campesterol | 37.58 | 0.71 |
| MOL009615 | 24-Methylenecycloartan-3beta,21-diol | 37.32 | 0.8 |
| MOL007063 | przewalskin a | 37.11 | 0.65 |
| MOL009617 | 24-ethylcholest-22-enol | 37.09 | 0.75 |
| MOL007061 | Methylenetanshinquinone | 37.07 | 0.36 |
| MOL001771 | poriferast-5-en-3beta-ol | 36.91 | 0.75 |
| MOL000358 | beta-sitosterol | 36.91 | 0.75 |
| MOL008173 | daucosterol_qt | 36.91 | 0.75 |
| MOL007121 | miltipolone | 36.56 | 0.37 |
| MOL000006 | luteolin | 36.16 | 0.25 |
| MOL002222 | sugiol | 36.11 | 0.28 |
| MOL007107 | C09092 | 36.07 | 0.25 |
| MOL009604 | 14b-pregnane | 34.78 | 0.34 |
| MOL007127 | 1-methyl-8,9-dihydro-7H-naphtho[5,6-g]benzofuran-6,10,11-trione | 34.72 | 0.37 |
| MOL007149 | NSC 122421 | 34.49 | 0.28 |
| MOL007049 | 4-methylenemiltirone | 34.35 | 0.23 |
| MOL009677 | lanost-8-en-3beta-ol | 34.23 | 0.74 |
| MOL009678 | lanost-8-enol | 34.23 | 0.74 |
| MOL007036 | 5,6-dihydroxy-7-isopropyl-1,1-dimethyl-2,3-dihydrophenanthren-4-one | 33.77 | 0.29 |
| MOL007143 | salvilenone Ⅰ | 32.43 | 0.23 |
| MOL007059 | 3-beta-Hydroxymethyllenetanshiquinone | 32.16 | 0.41 |
| MOL010234 | delta-Carotene | 31.8 | 0.55 |
| MOL007145 | salviolone | 31.72 | 0.24 |
| MOL007085 | Salvilenone | 30.38 | 0.38 |

Supplementary 1: 109 active compounds of *Salvia Miltiorrhiza (SM)*， and *Fructus lycii* (FL) were identified in in the TCMSP database.
