# Supplementary material for: Integrated network pharmacology and experimental validation to explore the potential pharmacological mechanism of Qihuang Granule and its main ingredients in regulating ferroptosis in AMD
Source: BMC Complement Med Ther. 2023 Nov 21;23:420. doi: 10.1186/s12906-023-04205-3 (PMC10664676; doi:10.1186/s12906-023-04205-3)
Supplement: Supplementary file 2 — Additional file 2. [file 12906_2023_4205_MOESM2_ESM.zip › WB.pptx]

## Slide 1
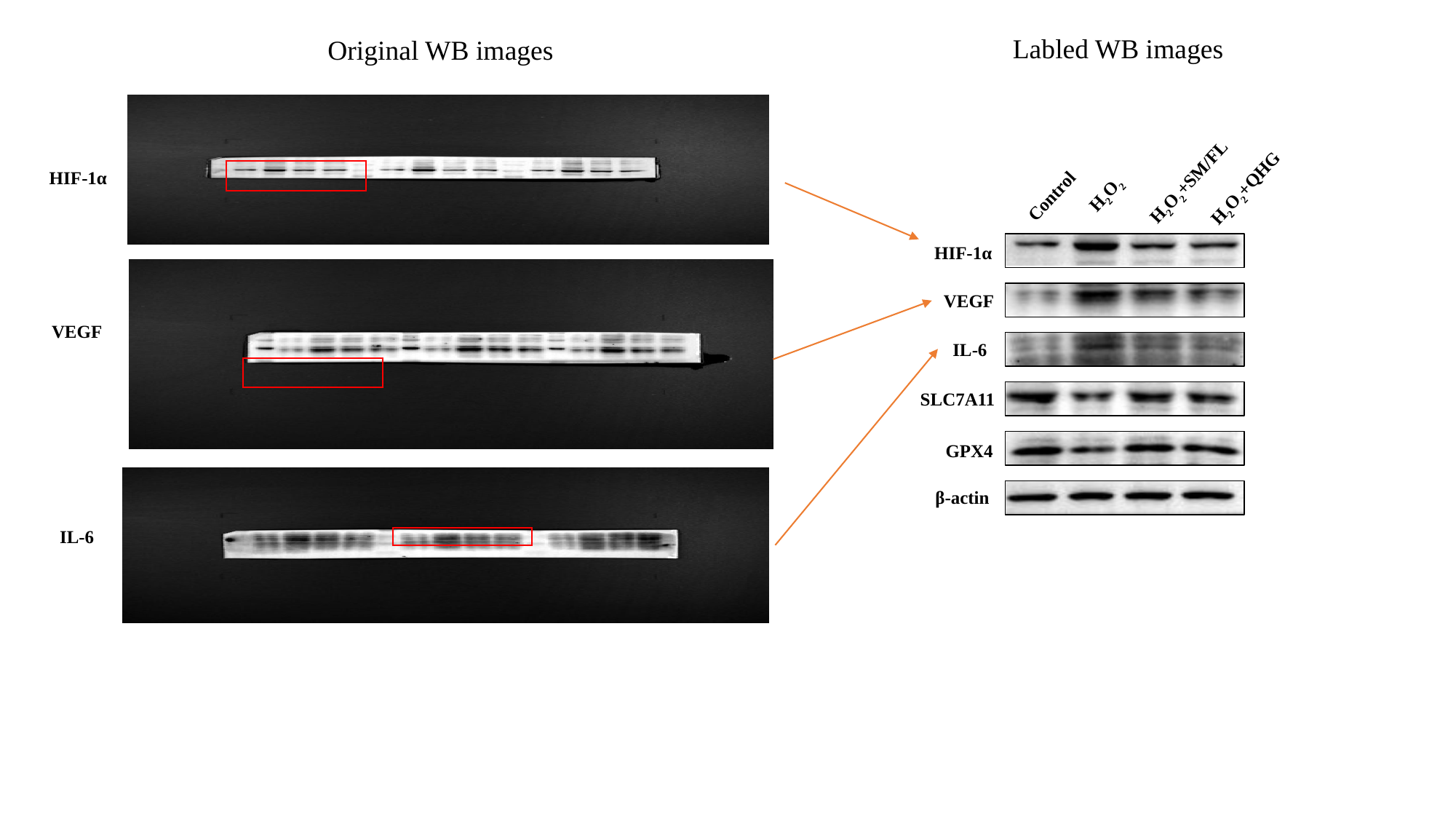

Labled WB images
Original WB images
H2O2
Control
H2O2+SM/FL
H2O2+QHG
VEGF
IL-6
SLC7A11
GPX4
β-actin
HIF-1α
HIF-1α
VEGF
IL-6

## Slide 2
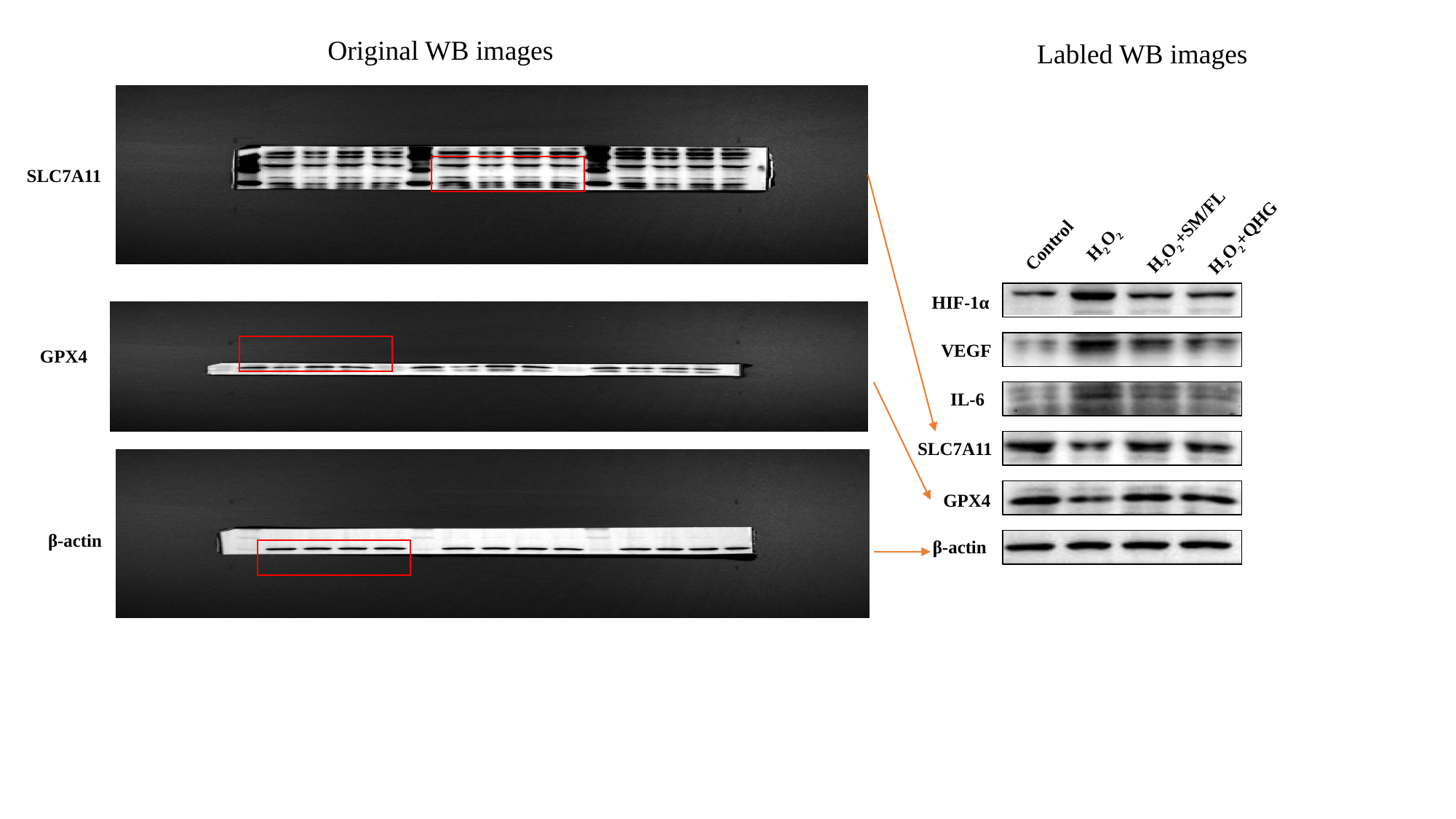

Original WB images
Labled WB images
H2O2
Control
H2O2+SM/FL
H2O2+QHG
VEGF
IL-6
SLC7A11
GPX4
β-actin
HIF-1α
SLC7A11
GPX4
β-actin
