# Supplementary figures and images for: Integrated network pharmacology and experimental validation to explore the potential pharmacological mechanism of Qihuang Granule and its main ingredients in regulating ferroptosis in AMD
Source: BMC Complement Med Ther. 2023 Nov 21;23:420. doi: 10.1186/s12906-023-04205-3 (PMC10664676; doi:10.1186/s12906-023-04205-3)

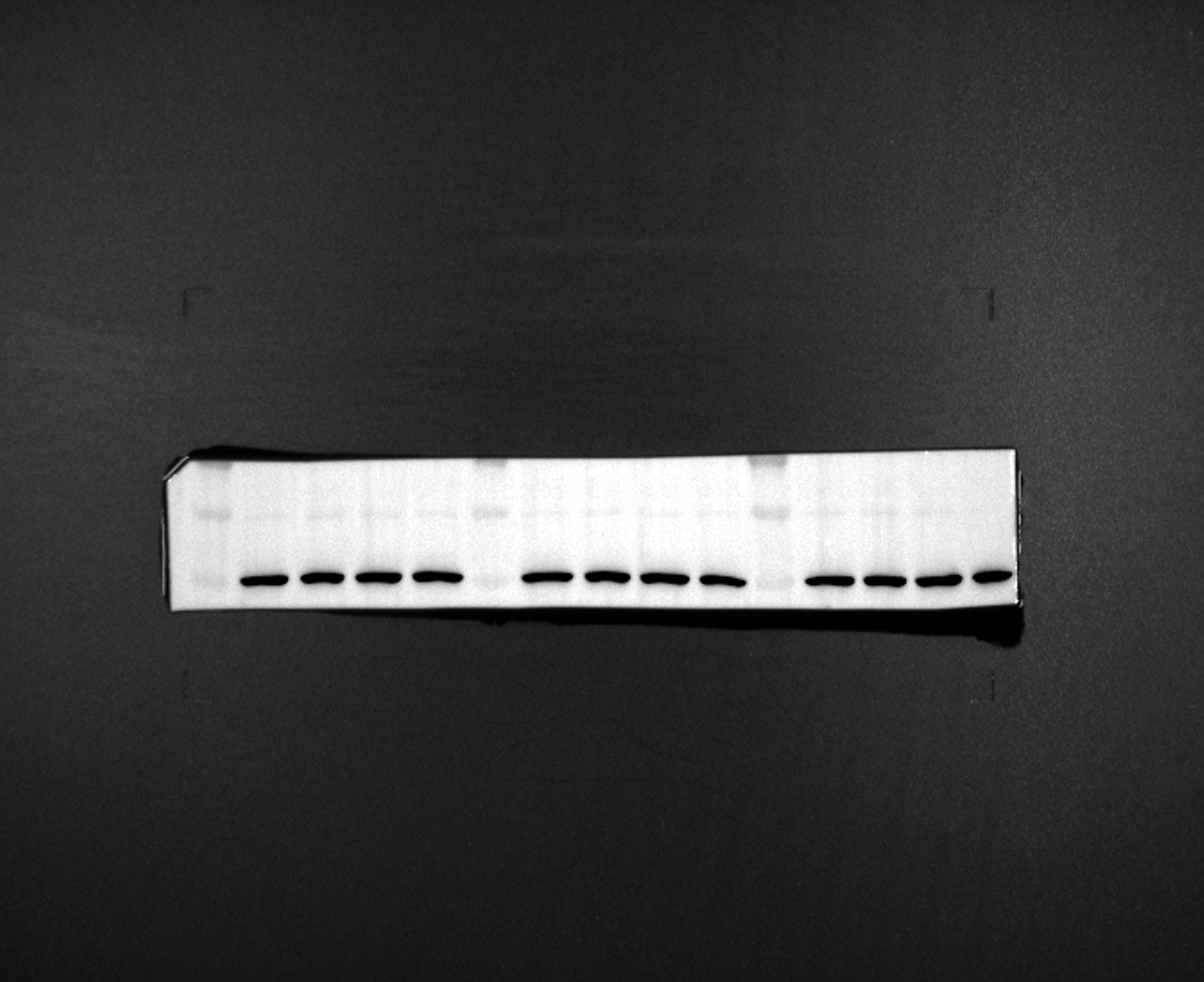

Supplement: Supplementary file 2 — Additional file 2. [file 12906_2023_4205_MOESM2_ESM.zip › actin.tif]

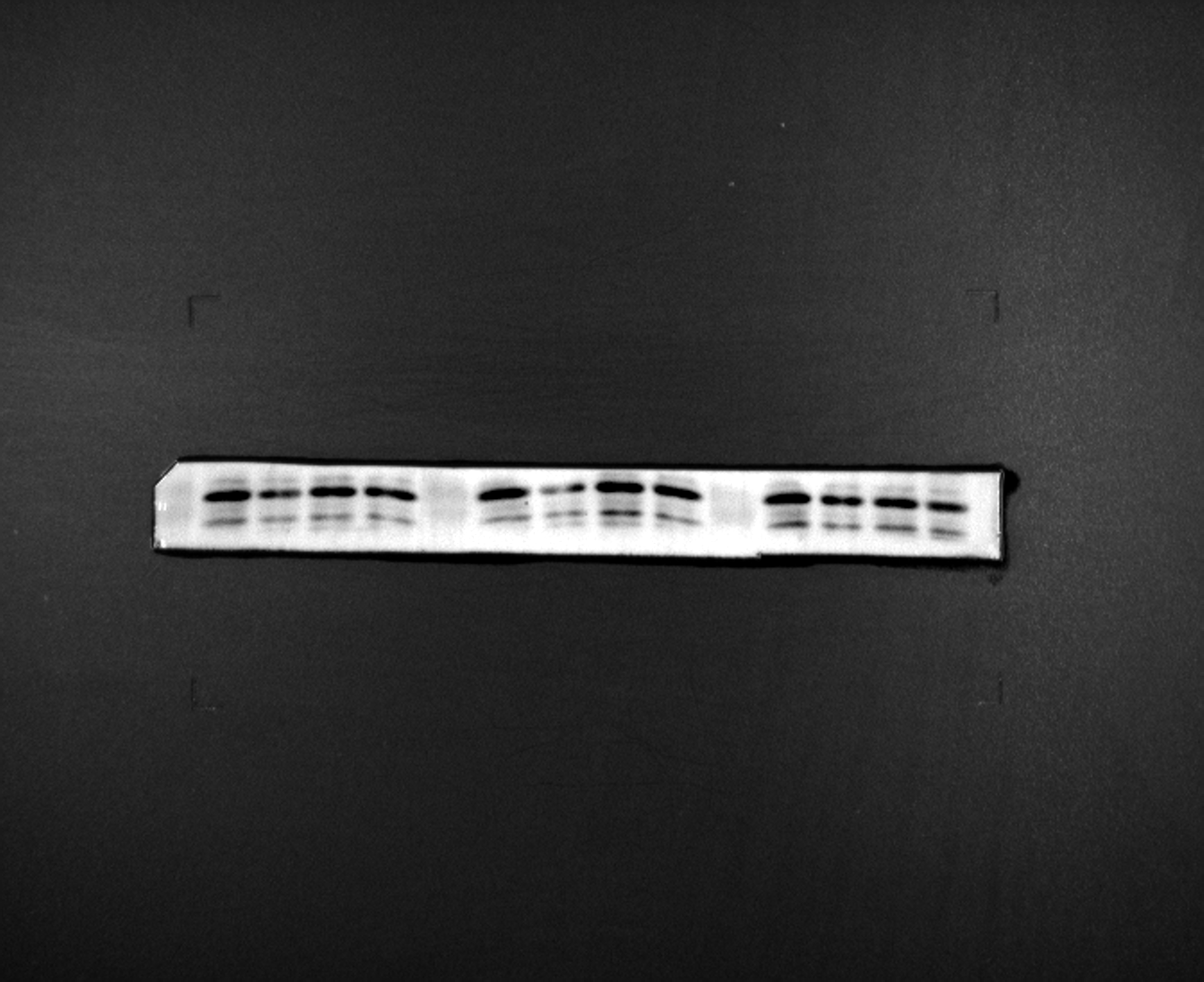

Supplement: Supplementary file 2 — Additional file 2. [file 12906_2023_4205_MOESM2_ESM.zip › GPX4.tif]

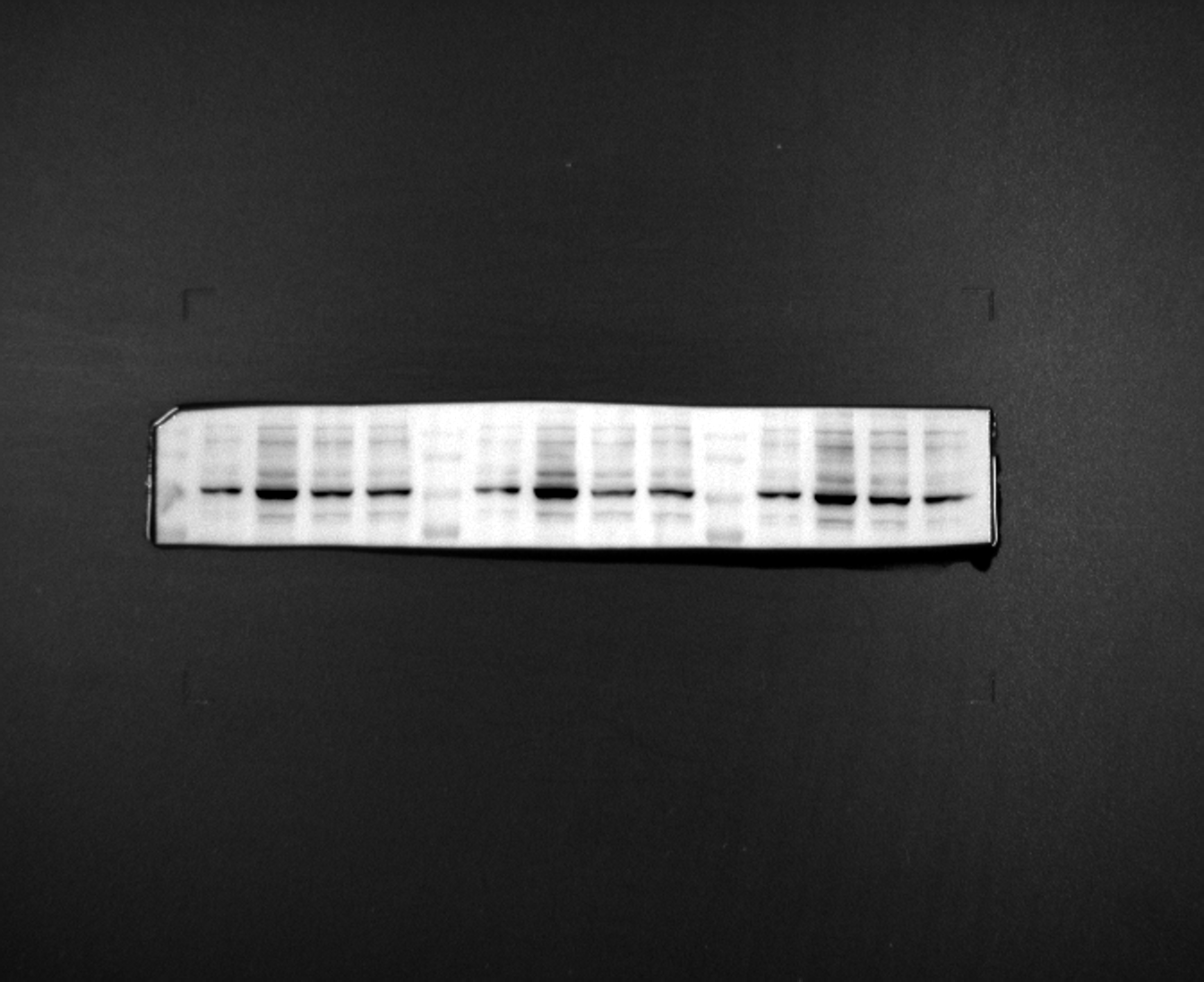

Supplement: Supplementary file 2 — Additional file 2. [file 12906_2023_4205_MOESM2_ESM.zip › HIF-a.tif]

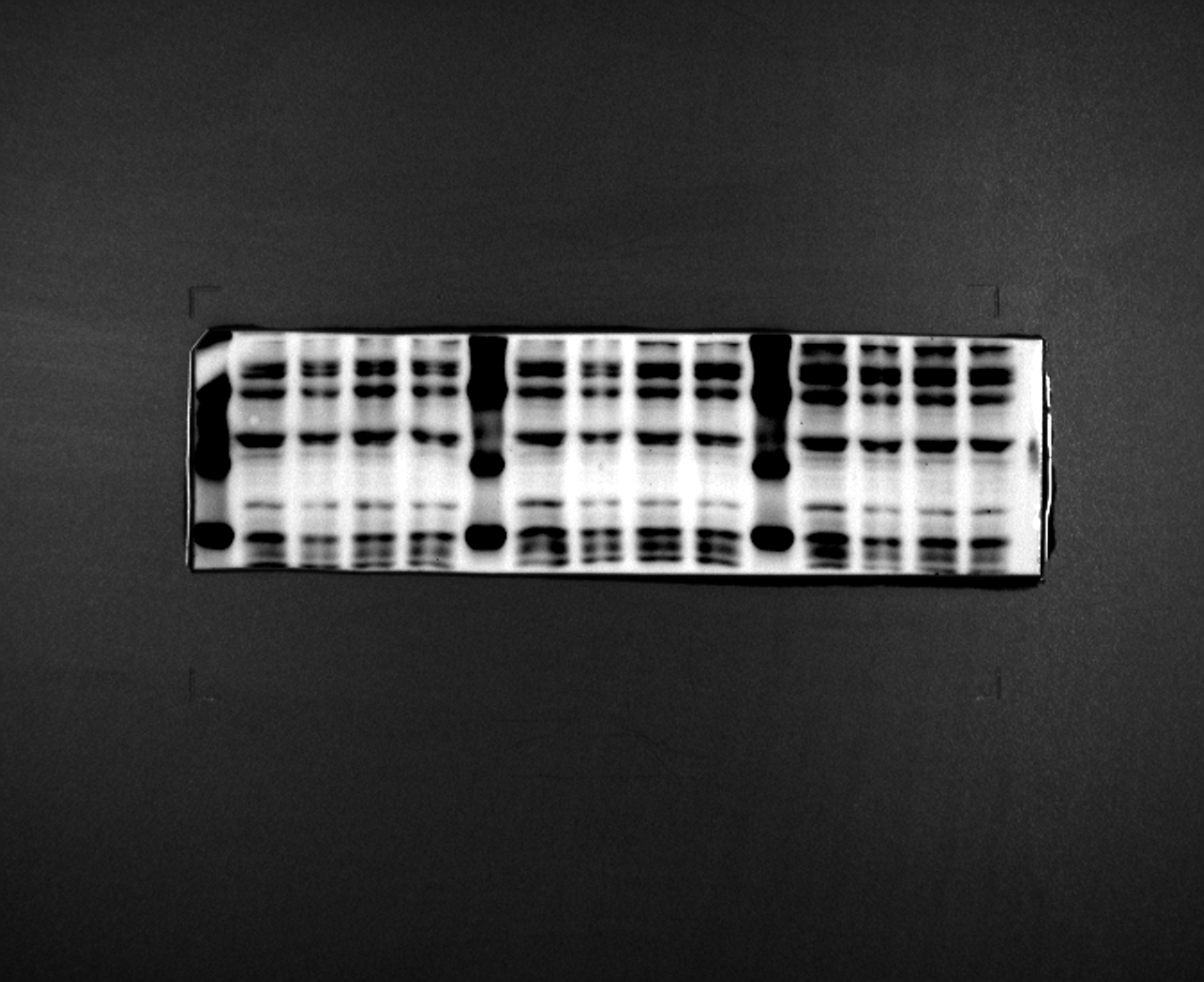

Supplement: Supplementary file 2 — Additional file 2. [file 12906_2023_4205_MOESM2_ESM.zip › SLC7A11.tif]

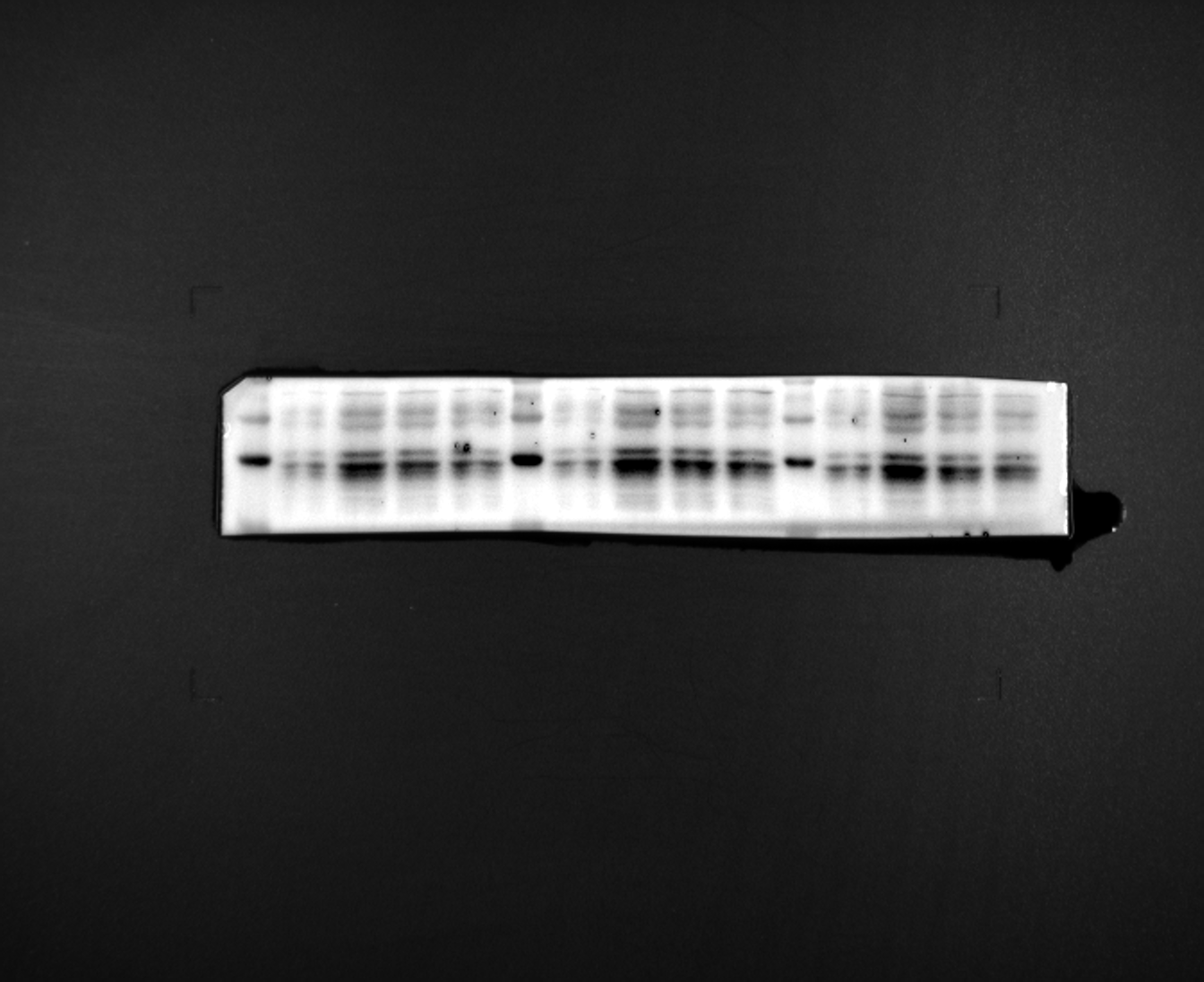

Supplement: Supplementary file 2 — Additional file 2. [file 12906_2023_4205_MOESM2_ESM.zip › VEGFa.tif]
